# Radiomics analysis of contrast-enhanced T1W MRI: predicting the recurrence of acute pancreatitis

**DOI:** 10.1038/s41598-022-13650-y

**Published:** 2023-02-16

**Authors:** Lingling Tang, Lin Ma, Yuying Chen, Yuntao Hu, Xinyue Chen, Xiaohua Huang, Nian Liu

**Affiliations:** 1 Department of Radiology, Affiliated Hospital of North Sichuan Medical College, No.63 Wenhua Road, Shunqing District, Nanchong, 637000 China; 2 Department of Hepatobiliary Surgery II, Affiliated Hospital of North Sichuan Medical College, No.63 Wenhua Road, Shunqing District, Nanchong, 637000 China

**Keywords:** Diseases, Health care, Medical research, Risk factors

## Abstract

To investigate the predictive value of radiomics based on T1-weighted contrast-enhanced MRI (CE-MRI) in forecasting the recurrence of acute pancreatitis (AP). A total of 201 patients with first-episode of acute pancreatitis were enrolled retrospectively (140 in the training cohort and 61 in the testing cohort), with 69 and 30 patients who experienced recurrence in each cohort, respectively. Quantitative image feature extraction was obtained from MR contrast-enhanced late arterial-phase images. The optimal radiomics features retained after dimensionality reduction were used to construct the radiomics model through logistic regression analysis, and the clinical characteristics were collected to construct the clinical model. The nomogram model was established by linearly integrating the clinically independent risk factor with the optimal radiomics signature. The five best radiomics features were determined by dimensionality reduction. The radiomics model had a higher area under the receiver operating characteristic curve (AUC) than the clinical model for estimating the recurrence of acute pancreatitis for both the training cohort (0.915 vs. 0.811, *p* = 0.020) and testing cohort (0.917 vs. 0.681, *p* = 0.002). The nomogram model showed good performance, with an AUC of 0.943 in the training cohort and 0.906 in the testing cohort. The radiomics model based on CE-MRI showed good performance for optimizing the individualized prediction of recurrent acute pancreatitis, which provides a reference for the prevention and treatment of recurrent pancreatitis.

## Introduction

Acute pancreatitis (AP) is an inflammatory disease characterized by edema and necrosis of glandular tissue, and it has become one of the most common acute abdominal conditions clinically^1^. Approximately 17 to 35% of AP patients may develop into recurrent acute pancreatitis (RAP)^2^. Some studies^3–5^ have suggested that AP, RAP, chronic pancreatitis, and pancreatic cancer may represent a disease continuum. A recurrent attack of AP is a potent risk factor for the progression of chronic pancreatitis^2^, thereby increasing the risk of pancreatic cancer. Therefore, it is essential to establish a stable and quantitative prediction model to predict the recurrence of AP, which can provide a precaution for potential recurrence patients and possibly help prevent cancer development.

The previous studies^6–10^ have primarily focused on exploring the clinical risk factors for predicting AP recurrence. However, simple clinical models show relatively low accuracy^7^ and lack stability and individual specificity due to the different research methods and clinical factors. Although imaging examinations show good values in the diagnosis of AP^11,12^, there are few radiological studies on RAP risk factors. These studies only explored the influence of pancreatic volume, CT severity index, and pancreatic necrosis on AP recurrence^13^. Meanwhile, existing prediction models only include the conventional morphological features of the images observed by doctors with the naked eye, so visualization of the potential subtle features reflecting the heterogeneity of the disease cannot be realized. Therefore, there is still a lack of accurate quantitative indicators to predict AP recurrence.

Radiomics, given the nature of texture analysis, could offer insight into the heterogeneity of lesions^14–17^. Therefore, the radiomics may can detect heterogeneous within etiological mechanisms and severity of acute pancreatic inflammation to indicate the prognosis and evolution of pancreatitis^18^. One previous study^7^ found that radiomics model based on computed tomography (CT) images has potential application value in predicting the recurrence of acute pancreatitis. While MRI, especially contrast-enhanced T1W MRI (observation of anatomy, early detection of necrosis or hemorrhage of pancreatic and peripancreatic)^19^, without radiation exposure, can provide more structural and functional information than CT. Therefore, whether radiomics analysis based on contrast-enhanced T1W MRI can be employed to predict RAP is an issue worth discussing.

To solve this problem, we performed radiomics analysis based on contrast-enhanced MR arterial late images from patients with an initial attack of AP. We built a radiomics model and compared it with the clinical model to evaluate prediction capability.

## Materials and methods

### Patients

The present study was approved by the Medical Ethics Committee of the Affiliated Hospital of North Sichuan Medical College and was exempted from informed consent requirements owing to its retrospective design (2020ER203-1). All the procedures performed in this study were in accordance with the Declaration of Helsinki (as revised in 2013).

**Figure 1:**
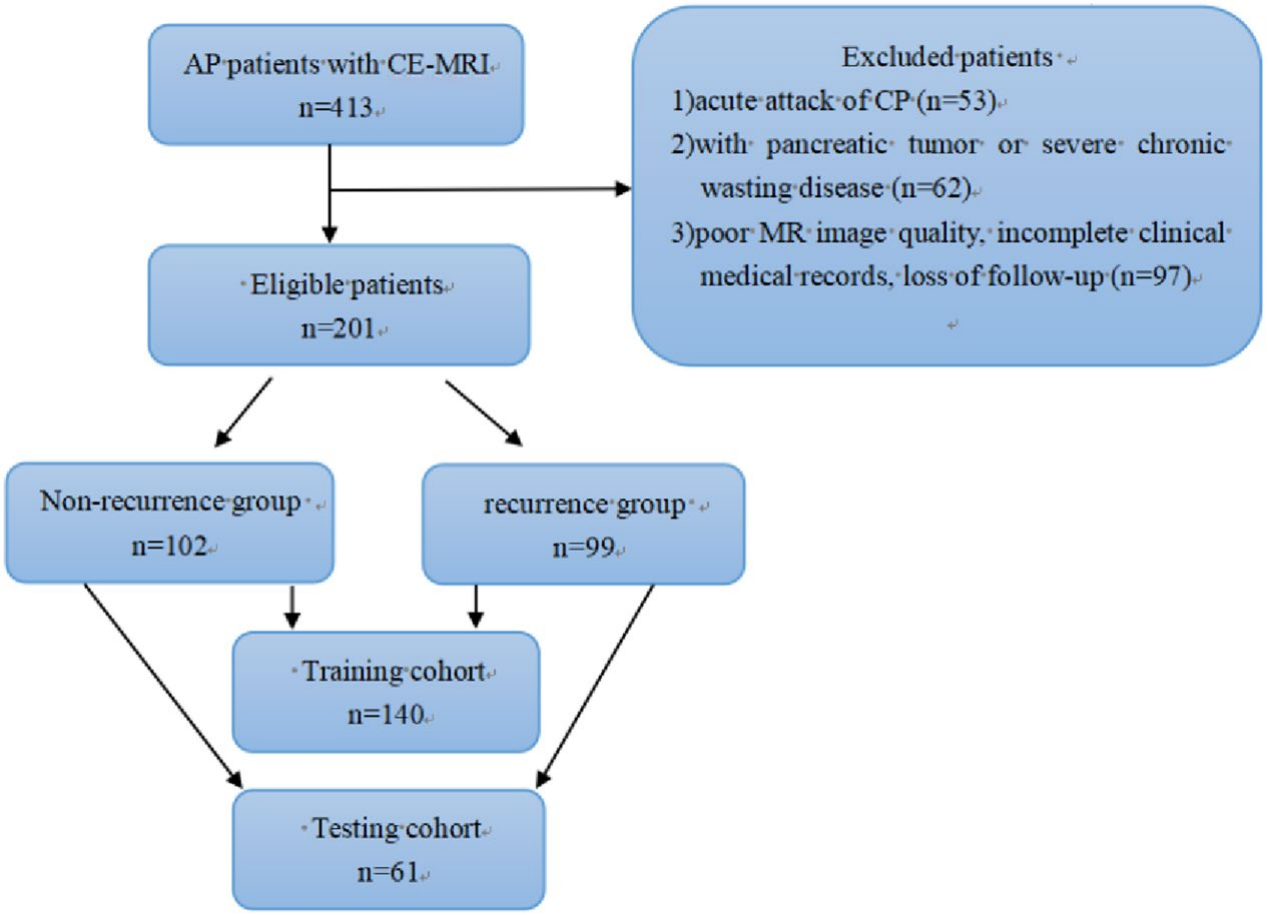
Flowchart of subject recruitment in this study. AP, acute pancreatitis; CE-MRI, contrast-enhanced MRI; CP, chronic pancreatitis.

The medical records of patients with AP treated at our hospital from January 2017 to December 2020 were consecutively reviewed, and follow up occurred through telephone or admission notes to record recurrence. In line with the inclusion and exclusion criteria, 212 patients were excluded and a total of 201 patients were recruited (Fig. 1). Based on the recurrence results during the follow-up period, the cases were split into a nonrecurrence group (n = 102) and a recurrence group (n = 99). The average time interval between the first attack and the second attack in the recurrence group was 21.9 ± 14.7 months. Using computer-generated random numbers in R software, the datasets were randomly split in a 7:3 ratio into a training cohort (n = 140, 71 in the nonrecurrence group, 69 in the recurrence group) and a testing cohort (n = 61, 31 in the nonrecurrence group, 30 in the recurrence group). Furthermore, class-relevant clinical characteristics of the patients were collected, including age and sex; history of alcoholism, smoking or hyperlipidemia; and disease characteristics such as severity, the MR severity index (MRSI) score, biochemical indices of pancreatitis (serum amylase, lipase, and pancreatic amylase levels), and the presence of biliary stones or local complications.

### Inclusion and exclusion criteria

Inclusion criteria: (1) hospitalized patients with their first attack of AP, and the diagnostic criteria of AP referred to the Atlanta Classification revised in 2012^20,21^; (2) plain MR scan and contrast-enhanced examination were performed within 7 days after onset; and (3) follow-up through phone calls or hospital records to verify recurrence during the follow-up period. The diagnostic standard of RAP^20^ includes two or more AP attacks and an interval between the two episodes of AP of at least 3 months, during which the patients reached the standard of recovery or near recovery.

The exclusion criteria were as follows: (1) patients with an acute attack of chronic pancreatitis; (2) patients with a pancreatic tumour or severe chronic wasting disease; and (3) patients with poor MR image quality (eg: respiratory motion artifacts caused by poor breath holding of patients) judged by the board certificated expert radiologist, incomplete clinical medical records, or loss of follow-up.

### MR scan equipment and scan parameters

All patients underwent an MR scan with a GE Discovery MR750 3.0 T, 32 channel body phased-array coil. The scan sequences were as follows. Axial single-shot fast spin-echo T2-weighted imaging, Axial 3D liver acquisition with volume acceleration-flexible (LAVA-Flex) T1-weighted imaging, and Axial contrast-enhanced LAVA-Flex T1-weighted imaging (TR = 3.8 ms, TE = 1.7 ms, slice thickness = 5 mm with no gap, matrix = 224 × 192, FOV = 36 cm × 36 cm). Contrast-enhanced scans were performed approximately 16–30 s (three arterial phases), 60 s (portal venous phase), and 120 s (delayed phase) after the injection of gadolinium (Gadobenate dimeglumine; Boleko Xinyi Pharmaceutical, Shanghai, China) administered intravenously at 2–3 mL/s, followed by a 10 mL normal saline solution flush. We scanned three arterial phases and selected the phase with better enhancement of abdominal aorta, proper hepatic artery, superior mesenteric artery and other branches as the late arterial images for analysis. All images are obtained from the picture archiving and communication system.

### Image analysis

**Figure 2:**
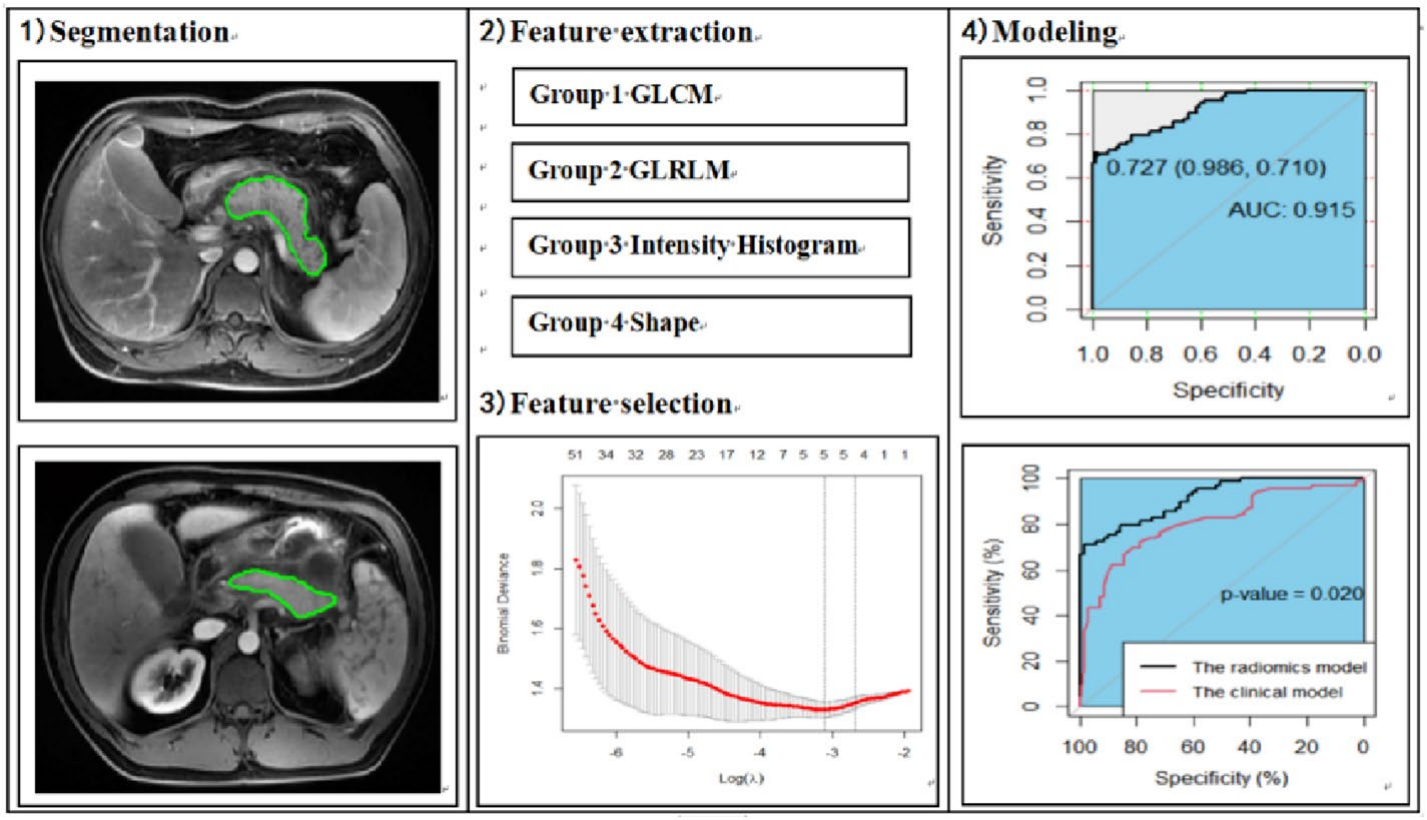
Flowchart of radiomics analysis. GLCM, gray-level co-occurrence matrix; GLRLM, gray-level run-length matrix.

Images of the contrast-enhanced late arterial phase were used for radiomics analysis (Fig. 2) because one study^22^ showed that pancreatic parenchymal enhancement in late arterial phase images was the best. Two radiologists who had 8 and 10 years of working experience in abdominal diagnosis (Reader 1 and Reader 2), without knowing the data of patients, drew the region of interest (ROI) of the pancreatic parenchyma, layer by layer, including the necrotic area of the pancreas, and avoided blood vessels and the common bile duct as much as possible. Image segmentation and radiomics feature extraction were carried out using IBEX^23^ (β1.0, http://bit.ly/IBEX_MDAnderson). Although we used the same equipment and scanning parameters, in order to further ensure the robustness and repeatability of radiomics features, we used the interpolation resampling method^7,24^ to process the image before extracting features and resample the voxel resolution of all images to 1 mm × 1 mm × 1 mm. A total of 428 radiomics features were extracted (see Supplemental Data), including the gray-level co-occurrence matrix (GLCM), gray-level run-length matrix (GLRLM), intensity histogram, and shape. All values of the features were calculated and obtained at the three-dimensional level. To eliminate the dimensions of the feature magnitudes and to ensure the reliability of the model, the preprocessing step of z-score normalization was applied to the feature data.

### Intrareader and interreader agreement

To evaluate the interreader agreement, Reader 1 and Reader 2 drew regions of interest independently and extracted features from the contrast-enhanced late arterial-phase images by selecting 50 patients randomly. To evaluate the intrareader agreement, Reader 1 sketched the ROI again one week later using the same method and extracted features and compared it with the first extracted features. Interreader and intrareader correlation coefficients (ICCs) > 0.75 were considered to have good consistency.

### Radiomics feature selection

To reduce the curse of dimensionality and avoid overfitting of the radiomics feature models, this study used the following methods for feature selection in the training cohort. First, the independent sample *t-*test or Mann–Whitney *U* test were applied to compare the features between the nonrecurrence group and the recurrence group for selecting the potentially important features. Then, the least absolute shrinkage and selection operator (LASSO) was used to reduce the dimension. Through the regularization process, the variables were selected, and the complexity was adjusted at the same time to obtain the optimal feature subset to improve the accuracy and repeatability of the radiomics prediction model. In this study, the regularization parameter (λ) was tuned, and tenfold cross-validation was used to select features. The best feature subset was obtained by using the principle of the minimum variance model.

### Establishment of the clinical model and radiomics model

The clinical characteristics with significant differences between the nonrecurrence group and recurrence group were used to construct the clinical model. The optimal radiomics features retained by dimension reduction were modelled by multivariable logistic regression. The area under the receiver operating characteristic curve (AUC), sensitivity, specificity, accuracy, precision, recall and F1-score were calculated to evaluate the predictive value of the model.

### Clinical use

The nomogram model was established by linearly integrating the clinically independent risk factor with the optimal radiomics signature, which could provide a quantitative tool for clinicians to individually predict RAP. The calibration curve was used to evaluate the consistencies between the actual recurrence rates and the nomogram-predicted probabilities of recurrence. The clinical benefit of using the model to guide clinicians in decision-making about treatment or intervention was evaluated by decision curve analysis (DCA).

### Statistics

Statistical analyses were conducted by using SPSS 25.0. Continuous variables were measured as the means or medians based on their distributions. The Kolmogorov–Smirnov method was applied to test the normality of the data. Independent sample *t*-tests and Mann–Whitney *U* tests were used for continuous variables, when appropriate. The chi-square test or Fisher’s exact test was used for categorical variables. Radiomics feature analysis and modelling were conducted using R (version 4.0.3, https://www.r-project.org/). The "*pROC*" installation package was used to draw the receiver operating characteristic (ROC) graph. The AUCs of the models were compared by the DeLong test. The differences were considered significant at *P* < 0.05.

## Results

### Clinical manifestations

The clinical manifestations of the cases in both the nonrecurrence group and recurrence group are listed in Table 1. Statistical differences were observed in sex, history of hyperlipidemia or smoking, local complications, and disease characteristics, such as MRSI and disease severity between the two groups (all *p* < 0.05). The proportion of males, a history of smoking, and the incidence of hyperlipidemia in the recurrence group were higher than those in the nonrecurrence group. However, the incidence of local complications and the MRSI in the nonrecurrence group were higher than those in the recurrence group. There were no significant differences between the two groups in age (*p* = 0.159), alcoholic aetiology (*p* = 0.056), biochemical indices of pancreatitis (all *p* > 0.05) or the presence of biliary stones (*p* = 0.759). A history of hyperlipidemia was an independent risk factor for AP recurrence, and the OR was 5.405 (95% CI 2.770–10.526). The clinical characteristics with statistical differences between the two groups were used to construct the clinical model.

**Table 1:** Clinical characteristics of the nonrecurrence group and recurrence group.

	Nonrecurrence group (n = 102)	Recurrence group (n = 99)	*P* value
Age (years)	50.02 ± 15.66	47.01 ± 14.51	0.159
Sex (male/female)	54/48	71/28	0.006*
Hyperlipidemia (n/%)	26/25.50	65/65.70	0.000*
Alcoholism (n/%)	32/31.40	44/44.40	0.056
Smoking (n/%)	29/28.40	48/48.50	0.003*
Serum amylase	602.85 (288.35–1379.75)	420.80 (288.35–1379.75)	0.052
Lipase	945.40 (282.03–1789.20)	689.00 (356.60–1340.80)	0.611
Pancreatic amylase	534.55 (201.70–1166.10)	411.00 (193.20–821.00)	0.133
Biliary stones (n/%)	36/35.30	37/37.40	0.759
Local complications (n/%)	73/71.60	48/48.50	0.001*
MRSI	5 (3–7)	4 (2–6)	0.004*
**Disease severity (n/%)**			0.018*
Mild	21/20.60	38/38.40	
Moderate	53/52.00	43/43.40	
Severe	28/27.50	18/18.20	

MRSI, MR severity index.

*P value < 0.05.

### Intrareader and interreader agreement, radiomics feature selection

**Figure 3:**
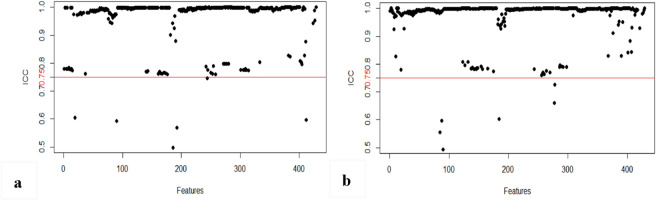
Consistency test of radiomics features. Evaluation of stability and consistency of radiomics features based on interreader (**a**) and intrareader (**b**) correlation coefficient (ICC), ICC > 0.75 (above the red line) indicates that the feature has good stability or inter and intrareader consistency.

**Figure 4:**
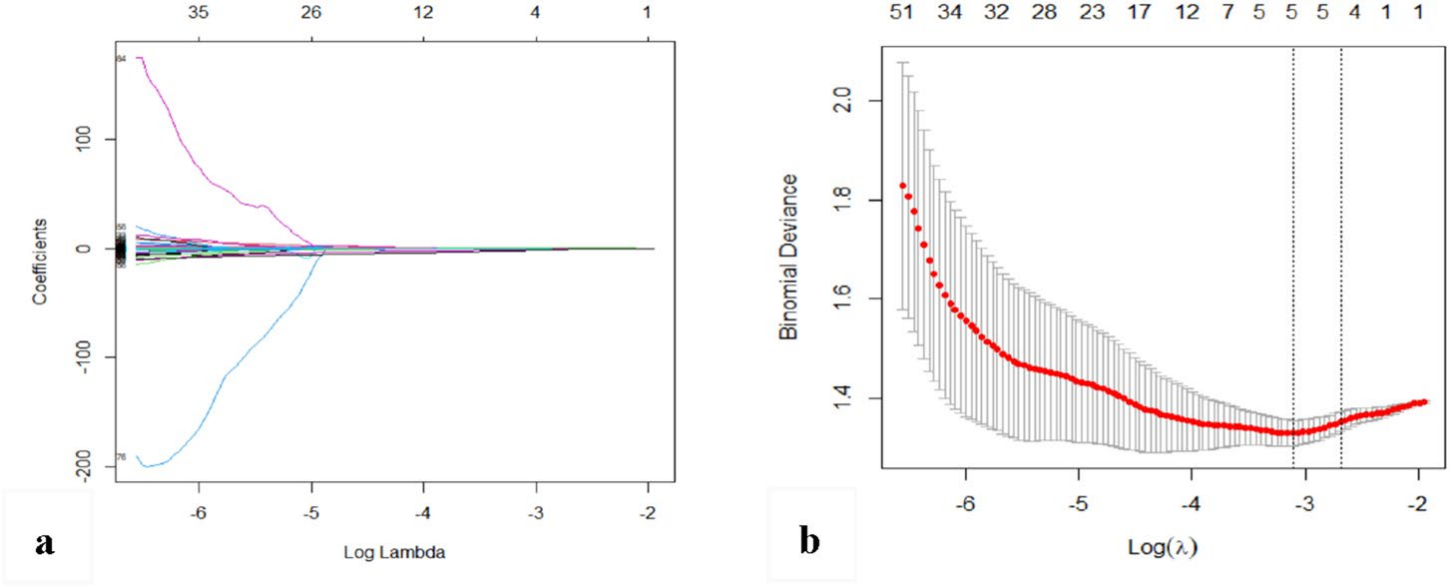
Feature selection using the least absolute shrinkage and selection operator (LASSO). (**a**) The coefficient distribution map of radiomics features and the features with nonzero coefficients were selected. (**b**) Adjustment parameter (λ) by tenfold cross validation. The dotted lines on the left and right represent the minimum variance principle and the most concise model principle, respectively. The minimum variance principle was adopted in this study.

There were 378 features with good consistency in the interreader agreement (mean ICC = 0.864) and 400 features in the intrareader agreement (mean ICC = 0.921). Finally, 66 features were excluded and 362 features were retained for dimensionality reduction (Fig. 3). One feature conformed to the normality test, and the independent sample *t*-test showed that the feature had a statistically significant difference between the nonrecurrence group and recurrence group. Among the remaining 361 features, the Mann–Whitney *U* test showed that 357 features had statistically significant differences between the two groups. A total of 358 features were included for LASSO regression (Fig. 4). After adjusting the parameter λ by tenfold cross-validation and adopting the principle of the minimum variance model, the optimal feature subset containing five features was finally obtained for model construction.

### Establishment of the clinical model and radiomics model

The logistic regression equation of clinical model was: Y = 1.83*X + 1.06 (Y represented the risk of recurrence, X represented hyperlipidemia; When the patient has hyperlipidemia, the variable was recorded as 1, otherwise recorded as 0). The logistic regression equation of radiomics model was: Y = 12.34 − 0.81*X_1_ − 8.35*X_2_ − 6.66*X_3_ (Y represented the risk of recurrence, X_1_ represented 45-1ClusterShade, X_2_ represented Surface Area Density, X_3_ represented Voxel Size).

**Figure 5:**
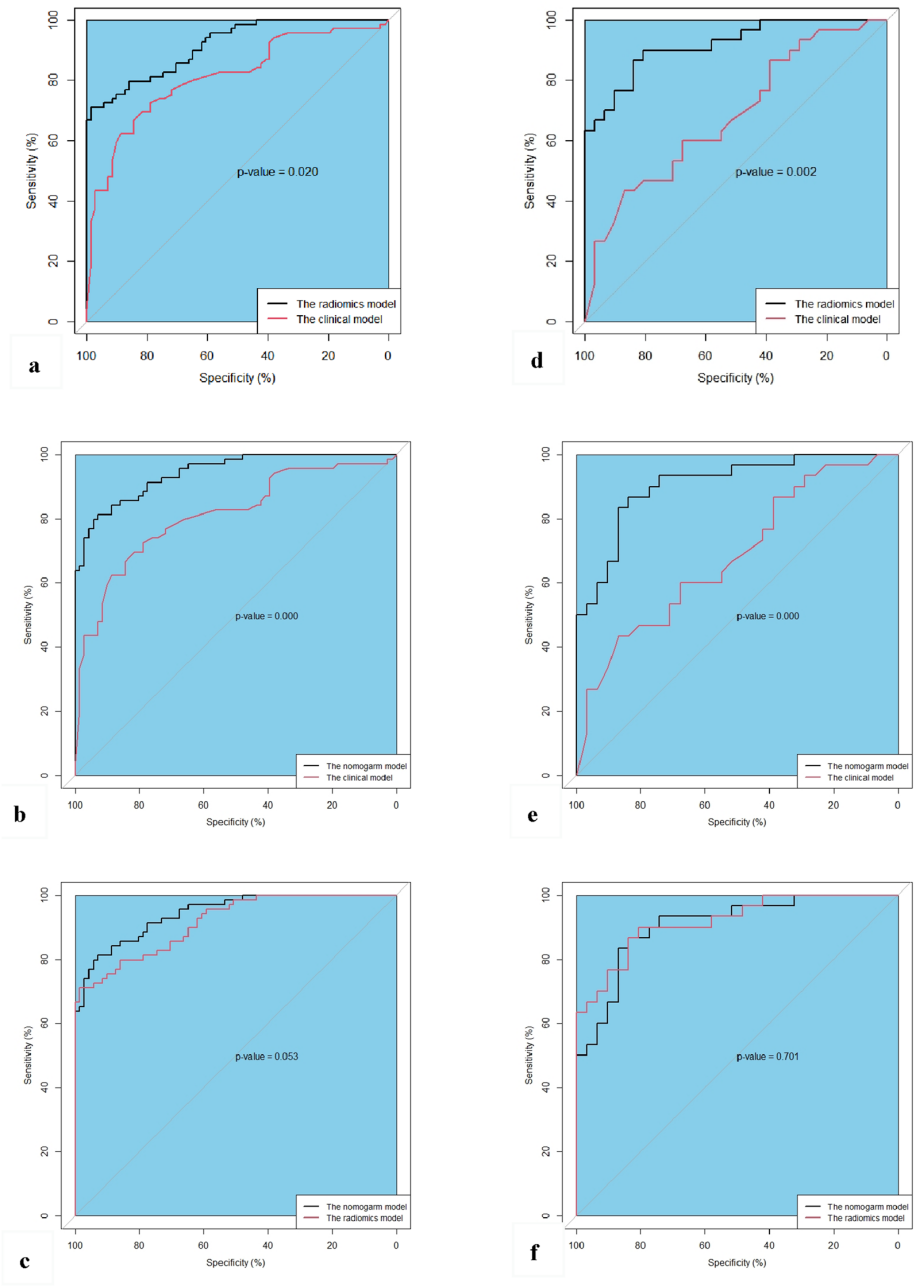
Comparison of the AUC among the three models in the training cohort (**a**–**c**) and testing cohort (**d**–**f**). AUC, area under the receiver operating characteristic curve.

In the training cohort, the radiomics model proved good performance for estimating the recurrence of acute pancreatitis, with an AUC of 0.915 (95% CI 0.871–0.958) and an accuracy of 82.9%. The AUC and accuracy of the clinical model were 0.811 (95% CI 0.738–0.883) and 74.3%, respectively. When comparing the AUCs between the models, the radiomics model displayed better results than the clinical model (*p* = 0.020). Meanwhile, good performance was observed in the testing cohort, with an AUC of 0.917 (95% CI 0.850–0.985) and an accuracy of 82.0%. The AUC and accuracy of the clinical model were 0.681 (95% CI 0.547–0.816) and 63.9%, respectively. Compared with the clinical model, the radiomics model showed a higher AUC (*p* = 0.002) (Fig. 5, Table 2).

**Table 2:** Performance of the three models.

	AUC (95% CI)	ACC (%)	Sensitivity (%)	Specificity (%)	PPV (%)	NPV (%)	F1
**Training cohort**							
Nomogram model	0.943 (0.909–0.977)	86.4	84.1	88.7	87.9	85.1	85.9
Radiomics model	0.915 (0.871–0.958)	82.9	79.7	85.9	84.6	81.3	82.1
Clinical model	0.811 (0.738–0.883)	74.3	73.9	74.6	73.9	74.6	73.9
**Testing cohort**							
Nomogram model	0.906 (0.833–0.980)	80.3	73.3	87.1	84.6	77.1	78.6
Radiomics model	0.917 (0.850–0.985)	82.0	76.7	87.1	85.2	79.4	80.7
Clinical model	0.681 (0.547–0.816)	63.9	60.0	67.7	64.3	63.6	62.1

AUC, area under the receiver operating characteristic curve; ACC, accuracy; PPV, positive predictive value; NPV, negative predictive value.

### Clinical use

**Figure 6:**
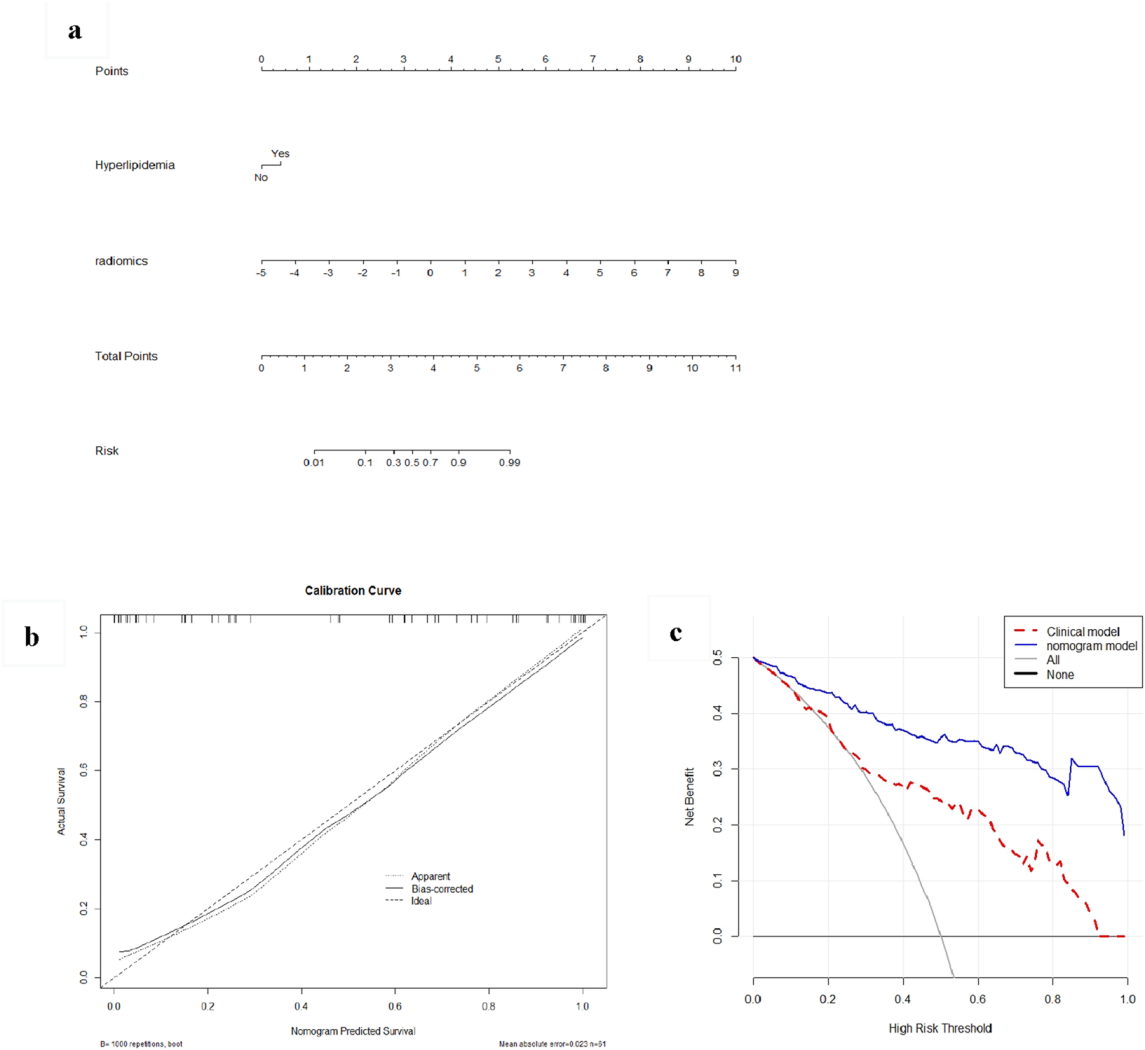
The nomogram model and decision curve analysis for predicting RAP. (**a**) Nomogram model for prediction RAP. (**b**) Calibration curve of the nomogram model. (**c**) Decision curve analysis for nomogram model. The *x*-axis represents the range of threshold probabilities. The y-axis measures the net benefit, as net benefit = sensitivity × prevalence − (1 − specificity) × (1 − prevalence) × *w*, where *w* was the odds in the threshold probabilities. The blue curve represents the nomogram model, and the red curve represents the clinical model. RAP, recurrent acute pancreatitis.

The nomogram model showed good performance, with an AUC of 0.943 (95% CI 0.909–0.977) in the training cohort and 0.906 (95% CI 0.833–0.980) in the testing cohort (Table 2). The risk of recurrence could be calculated from the total points. The calibration curve revealed that the predicted probability of recurrence could reflect the real risk calculated from our dataset by classifying patients with similar risks into groups. DCA revealed that if the threshold probability was above 0.02, applying the nomogram model to predict the recurrence of AP was of increased clinical net benefit and more beneficial than the clinical model (Fig. 6).

## Discussion

With the improvement of people's living standards and changes in dietary habits, the recurrence rate of AP increases year by year. In addition, 50% of RAP patients have gene mutations (such as repeated inflammatory stimulation that can lead to Kras gene mutations)^25^, which will increase the risk of chronic pancreatitis and pancreatic cancer and seriously affect the quality of patient's life^26^. Therefore, RAP is attracting increasing attention from clinicians and researchers. Although some clinical risk factors for RAP have been identified, it is still difficult to accurately determine the course of the disease in the future. Therefore, it is necessary to find a method that can quantitatively predict the possibility of AP recurrence to guard against the occurrence of RAP effectively.

In this research, we developed and verified an MRI-based radiomics model, with a high accuracy of 82.9%, to quantitatively predict AP recurrence. A radiomics study^7^ based on contrast-enhanced CT was similar to our results, which suggested that radiomics may have broad application prospects in alerting first-episode AP patients to their risk of recurrence. The radiomics model in our study was potent in its prediction ability, which may be due to the following three aspects. First, there may be subtle alterations in the pancreatic parenchyma after the first episode of AP^27,28^, and the radiomics features extracted from the image could reflect the quantitative information that cannot be identified by the unaided eye^29^. Our results found that the radiomics features (45-1ClusterShade, Surface Area Density, and Voxel Size) could predict the risk of RAP. Cluster shade is a measure of heterogeneity (asymmetric in three-dimensional space)^30,31^. Surface area density and voxel size describe the shape and geometric features of the three-dimensional ROI^32^. The higher the absolute value of cluster shade (when the degree was 45° and the step size was 1, the value was negative), or the smaller the value of surface area density or voxel size, the easier it was to relapse. This finding is consistent with the necrosis-fibrosis hypothesis that RAP could lead to progressive obstruction of acinar cell complex and atrophy of acinar cell^13^. Secondly, the variation in scanning parameters had a significant influence on the repeatability of imaging features^31,33^. However, our data were acquired by the same machines and parameters, and thereby high repeatability and accuracy were obtained. Finally, optimization of feature selection may improve the stability of the model. The LASSO regression algorithm^34,35^ was the most commonly used feature selection method in previous studies. It was suitable for small samples and extensive feature collection data analysis. By tenfold cross-validation and adjustment of the regularization parameters (λ), it could prevent the overfitting phenomenon and ensure the model's reliability. Therefore, the clinical application of radiomics is prospective in predicting the recurrence of AP.

Another significant result of our study was that the radiomics model had a higher AUC than the clinical model to predict the recurrence of AP, indicating that the simple clinical model has limited value in predicting RAP. Statistical differences were observed in sex, history of smoking, local complications, and characteristics, such as MRSI and disease severity between the two groups, and the history of hyperlipidemia was clinically independent risk factor for RAP. Previous studies^7,8,36,37^ have confirmed the above findings of our study. A previous study^37^ demonstrated that the recurrence rate of AP caused by hyperlipidemia was higher, with reported rates of approximately 30.1–44.2%. Additionally, a previous study^7^ showed that alcohol consumption and younger age were risk factors. However, no significant difference was found in alcoholism and age between the two groups in our study. The small size and single-center sample may have contributed to these results. In any case, the radiomics model is superior to the simple clinical model, which enables a new approach for predicting the recurrence of AP.

Moreover, we constructed a nomogram based on the history of hyperlipidemia and radiomics signature, in which the recurrence risk can be obtained quantitatively by a simple addition operation^2^. The nomogram is conducive to personalized prediction for patients. In addition, DCA^38,39^ was used to evaluate the net clinical benefit of the model. The AUC metric focuses only on the precision of the prediction of the model, so it does not tell us whether the model can be applied in clinical practice or which of several models is desirable^38^. However, DCA, which combines the consequences, can tell us whether the net clinical benefit of a model is sufficient or which of several models is preferable. In our study, DCA indicated that if the threshold probability was above 0.02, applying the nomogram model to predict the recurrence of AP had an increased net clinical benefit and was more helpful than the clinical model. This suggested that radiomics was of value in clinical practice and had broad application prospects.

Several limitations existed in this research. First, the sample size was relatively small, and our study was a single center, which may lead to selection bias of patients and imaging methods. Therefore, further multicentre and large sample studies are needed in the future. Second, a single-phase or sequence of MR images was selected for radiomics analysis, which may result in the loss of some useful information reflecting the disease. Thus, our future research aims to combine multiple phases or sequences to construct a more comprehensive and accurate model.

In summary, a radiomics model based on MR contrast-enhanced late arterial-phase images can be used to predict the recurrence of AP noninvasively and quantitatively. Both the radiomics and nomogram models showed better predictive performance than the clinical model for the recurrence of acute pancreatitis, and the nomogram model tended to be better than the radiomics model. Meanwhile, the nomogram model and DCA could guide clinicians in individualized decision-making about treatment or intervention for potential recurrence patients.

## Supplementary Information

Supplementary Information.

## Data Availability

The datasets generated and analysed during the current study are not publicly available due to our need to expand the sample size and further research based on this datasets, but are available from the corresponding author on reasonable request.
