# Supplementary material for: Radiomics analysis of contrast-enhanced T1W MRI: predicting the recurrence of acute pancreatitis
Source: Sci Rep. 2023 Feb 16;13:2762. doi: 10.1038/s41598-022-13650-y (PMC9935887; doi:10.1038/s41598-022-13650-y)
Supplement: Supplementary file 1 — Supplementary Information. [file 41598_2022_13650_MOESM1_ESM.pdf]

Radiomics analysis of contrast-enhanced T1W MRI: Predicting the recurrence of acute pancreatitis

Lingling Tang[1#], MS; Lin Ma[2#], MS; Yuying Chen[1], MS; Yuntao Hu[1], MS;

Xinyue Chen[1], MD; Xiaohua Huang[1*], PhD; Nian Liu[1*], MD

Supplemental Data

Table. The particular of radiomics features

Group	No.	Radiomics features
GLCM	N1-N330	Each GLCM feature is generated in five different degrees (-333°, 0°, 45°, 90°, 135°) and three different steps (1, 4, 7). Thus, each GLCM feature generates 15 sub-features and there are 330 features in total: Auto Correlation, Cluster Prominence, Cluster Shade, Cluster Tendendcy, Contrast, Correlation, Difference Entropy, Dissimilarity, Energy, Entropy, Homogeneity 1, Homogeneity 2, Information Measure Corr 1, Information Measure Corr 2, Inverse Diff Moment Norm, Inverse Diff Norm, Inverse Variance, Max Probability, Sum Average, Sum Entropy, Sum Variance, Variance
GLRLM	N331-N363	Each GLRLM feature is generated in three different degrees (-333°, 0°, 90°) and one step (1). Thus, each GLRLM feature generates 3 sub-features and there are 33 features in total: Gray Level Nonuniformity, High Gray Level Run Empha, Long Run Emphasis, Long Run High Gray Level Empha, Long Run Low Gray Level Empha, Low Gray Level Run Empha, Run Length Nonuniformity, Run Percentage, Short Run Emphasis, Short Run High Gray Level Empha, Short Run Low Gray Level Empha
Intensity histogram	N364-N411	Inter Quartile Range, Kurtosis, Mean Absolute Deviation, Median Absolute Deviation, Percentile, Percentile Area, Quantile, Range, Skewness
Shape	N412-N428	Compactness 1, Compactness 2, Convex, Convex Hull Volume, Convex Hull Volume 3D, Mass, Max 3D Diameter, Mean Breadth, Number of Voxel, Orientation, Roundness, Spherical Disproportion, Sphericity, Surface Area, Volume, Surface Area Density, Voxel Size

GLCM, gray-level co-occurrence matrix; GLRLM, gray-level run-length matrix.
